# Serum Zinc Ion Concentration Associated with Coronary Heart Disease: A Systematic Review and Meta-Analysis

**DOI:** 10.1155/2022/4352484

**Published:** 2022-10-06

**Authors:** Heyu Meng, Jianjun Ruan, Yanqiu Chen, Zhaohan Yan, Xin Meng, Xiangdong Li, Jinsha Liu, Cuiying Mao, Ping Yang

**Affiliations:** ^1^Department of Cardiology, China-Japan Union Hospital of Jilin University, Changchun 130033, China; ^2^Jilin Provincial Engineering Laboratory for Endothelial Function and Genetic Diagnosis of Cardiovascular Disease, Changchun 130033, China; ^3^Jilin Provincial Molecular Biology Research Center for Precision Medicine of Major Cardiovascular Disease, Jilin Provincial Cardiovascular Research Institute, Changchun 130033, China; ^4^Jilin Provincial Precision Medicine Key Laboratory for Cardiovascular Genetic Diagnosis, Changchun 130033, China

## Abstract

**Aim:**

Coronary heart disease is a major cause of mortality in developed and developing countries. Changes in the trace element concentration in the human body are one of the main reasons for the transition of the human body from a healthy to a diseased state. In this meta-analysis, we have studied the relationship between the reduction in serum zinc ion concentration and coronary heart disease.

**Methods:**

We used PubMed and Cochrane (as of June 30, 2021) databases for the literature search. Per the requirements of this systematic review, case-control studies involving serum zinc ion concentration and coronary heart disease were searched, and the quality of the included studies was evaluated before the meta-analysis.

**Results:**

A total of 3,981 cases were found across seven articles. The standard mean deviation (SMD) of serum zinc ion concentration was −0.22 [−0.28, −0.15], *z* = 6.52, and *P* < 0.05 indicated that the difference was statistically significant. The forest plot results show that *I*^2^ = 34% < 50%, and the *Q* test showed *P*=0.17 > 0.1. These results suggest a lack of heterogeneity among the selected articles. Results from the funnel chart indicated that this study was free from publication bias.

**Conclusion:**

The results of this meta-analysis reveal that a decrease in serum zinc ion concentration is related to the occurrence of coronary heart disease. Clinically, monitoring the serum zinc ion levels is proven to be of great significance for patients with coronary heart disease.

## 1. Background

Coronary artery disease (CHD) is the main cause of morbidity and mortality in developed countries [1, 2]. Although in the past 20 years, the median age percentage of patients who succumbed to CHD has decreased by 22% worldwide, CHD is still the leading cause of death in the world [2]. Although the mortality rate is declining [1–3], it is still the leading cause of hospitalization and death in the UK and worldwide [1]. The mortality rate of CHD in developing countries has also been showing an increasing trend over the years [4]. CHD caused 8.1 million deaths in 2013, accounting for 14.8% of global deaths [5]. From 1990 to 2013, CHD was the leading cause of human death worldwide [5]. A German study showed that early detection and timely treatment can increase the survival rate of patients with circulatory blocks by 40% [6], suggesting that early detection and treatment are still key in treating not just CHD but also other diseases.

Changes in the trace element concentration in the body are considered to be the main factor leading to the transition of the human body from a healthy to a diseased state [7–10]. Trace elements, especially zinc ions, are likely involved in the pathogenesis of CHD [11–13]. Zinc is an important element in more than 70 enzymes, including superoxide dismutase and glutathione peroxidase. Zinc can be a cofactor of Cu-Zn superoxide dismutase (Cu, Zn-SOD) and subsequently aid in treating CHD. Zinc ions participate in the regulation of various cellular metabolic activities, including the metabolism of different proteins, lipids, and carbohydrates in the body [14–16]. Most importantly, zinc exerts antioxidant and anti-inflammatory effects [17, 18]. An increase in the zinc ion concentration improves the antioxidant capacity of cells and ensures the secretion of a sufficient amount of NO to maintain normal endothelial function.

Based on the potential relationship between zinc ions and the occurrence of CHD and previous studies on zinc ions and CHD [16], we hypothesized that the decrease in serum zinc ion levels is related to the occurrence of CHD. To that end, we attempted to determine the relationship between serum zinc ion levels and CHD through articles on serum zinc ion concentration and CHD published in the past 10 years.

## 2. Methods

### 2.1. Search Strategy

We strictly followed the guidelines laid down for systematic reviews and meta-analysis (PRISMA) [19]. We used the PubMed and Cochrane databases for literature searches. The search keywords were used either singly or in a combination and included subject words and synonym words identified using MeSH. The subject word and synonym word for zinc ion is Zinc. The subject keyword of coronary heart disease is coronary disease, and the synonym words are coronary diseases; disease, coronary; diseases, coronary; coronary heart disease; coronary heart diseases; disease, coronary heart; diseases; coronary heart; heart disease, coronary; heart diseases, coronary. At the same time, we limited the scope of the search to reports published in English, and there was no limit to the time of publication of the literature. Before the final analysis, we once again perused and inspected the quality of the literature to ensure that only studies that met the review criteria were included. For example, in PubMed, the retrieval relationship between synonym words was “OR,” and the retrieval relationship between subject words and synonym words was “AND.”

### 2.2. Inclusion and Exclusion Criteria

**Figure 1:**
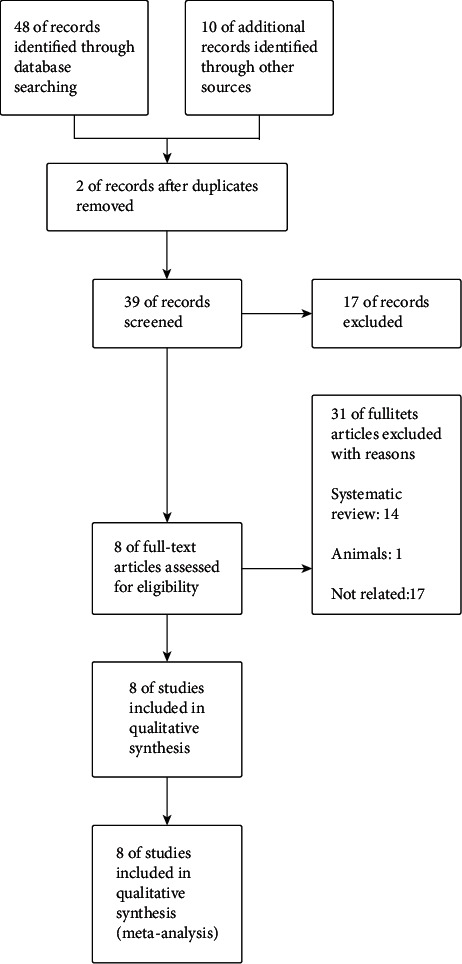
Flowchart for article screening.

The articles retrieved and selected were independently screened by two authors (HM, JR) based on the title, abstract, and full text. In addition, the points of disagreement were resolved through discussion. The inclusion criteria were research related to the topic, with data, including the average value of zinc concentration and its standard deviation. The exclusion criterion was repeated studies, review or meta-analysis, animal experiments, undetected zinc ion concentrations, and unstable studies (the unstable studies are the ones with no strict selection criteria, unreasonable statistics, and exaggerated conclusions). See Figure 1 for details.

### 2.3. Quality Evaluation

The quality and data extracted from each study were evaluated per the Methodological index for nonrandomized studies (MINORS) guidelines. All data were independently extracted by two reviewers (HM and JR). Disagreements were resolved by involving a third unbiased reviewer. The extracted data included the name of the first author, year of publication, country/region, study design, sample size, and baseline characteristics, and whether the gold standard was applied for disease detection.

### 2.4. Statistical Analysis

A meta-analysis was performed to comprehensively analyze different studies. The standard mean deviation (SMD) and corresponding 95% confidence interval (CI) were used to evaluate the difference between serum zinc ion and CHD in the selected articles. For determining heterogeneity, the random-effects or fixed-effects models were used. The *I*^2^ test was used to evaluate the statistical heterogeneity between the studies, where the values of *I*^2^ > 25% and 50% were regarded as moderate and high heterogeneity, respectively. In Statistics, *P* < 0.05 was considered statistically significant. In addition, we performed a sensitivity analysis to assess the robustness of the results. We also used a funnel chart to assess publication bias [20]. All analyses were performed using Review Manager 5.3 (Copenhagen, the Nordic Cochrane Centre, the Cochrane Collaboration).

## 3. Results

### 3.1. Characteristics of Each Study

In Table 1, (1) the purpose of the study is clearly given; (2) the consistency of the included patients; (3) the collection of expected data; (4) the endpoint indicators appropriately reflect the purpose of the research; (5) the objectivity of the evaluation of the endpoint indicators; (6) whether the follow-up time is sufficient; (7) the loss to follow-up rate is less than 5%; (8) whether the sample size is estimated; (9) whether the selection of the control group is appropriate; (10) whether the control group is synchronized; (11) whether the baselines between the groups are comparable; and (12) whether the statistical analysis is appropriate. Scoring method: 0 point means not reported; 1 point means reported but insufficient information; 2 points mean reported and with sufficient information. Articles with a score of 0–8 are classified as low-quality articles, 9–16 as medium-quality articles, and 17–24 as high-quality documents. The MINORS quality evaluation form denotes literature with a score of fewer than 12 points as excluded from the meta-analysis. Scoring was performed independently by two researchers. Inconsistent scoring results were resolved through discussion or consultation with an independent third party until an agreement was reached. The seven articles included in the study had scores of 15–21 points, suggesting them all to be medium- and high-quality articles.

### 3.2. Heterogeneity Test

The seven articles included in this study were tested for heterogeneity, if *I*^2^ = 34% < 50%, and *Q* test *P*=0.17 > 0.1. These results suggest a lack of heterogeneity between the selected articles in this study, and the fixed-effects model was chosen for the meta-analysis. To ensure the accuracy and stability of the study, we conducted a sensitivity analysis.

### 3.3. Sensitivity Analysis

A sensitivity analysis was carried out on the seven articles included in this study. One article was removed at a time, and none of them interfered with the results of this meta-analysis, indicating that the study had good stability. See Table 2 for details.

### 3.4. Meta-Analysis of Fixed Effects

**Figure 2:**
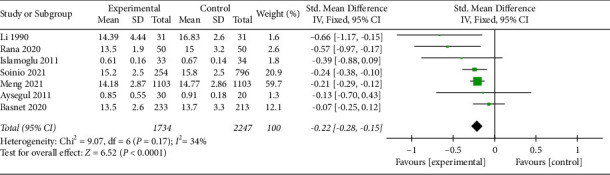
Forest diagram.

The SMD value of the seven studies was −0.22, 95% confidence interval was −0.28∼−0.15, *z* = 6.52, and *P* < 0.05, which was statistically significant. These results suggest that serum zinc ion concentration was related to CHD. The results are shown in the forest diagram (Figure 2).

### 3.5. Bias Test

**Figure 3:**
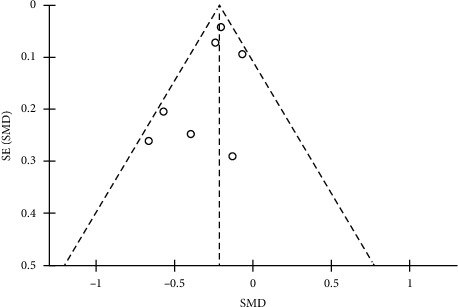
Funnel chart the funnel plot is symmetrical on both sides and there is no publication bias.

A funnel chart was constructed to investigate whether there was a publication bias in this study; the symmetry in the funnel chart indicated no publication bias, as observed in Figure 3.

## 4. Discussion

Through this meta-analysis, we aimed to correlate the serum zinc ion concentration to the occurrence of CHD by including studies that compared the serum zinc ion concentration in patients with CHD to that in the controls. A total of seven articles met our criteria. The results showed that the serum zinc ion concentration in CHD patients was higher than that of the control group, suggesting that the serum zinc ion level has a potential impact on the occurrence of CHD.

Medical-physiological studies have shown a correlation between trace element content and CHD [27–30]. He et al. proposed that an increase in zinc ion concentration can significantly reduce high-density lipoprotein (HDL) levels and increase triglyceride (TG), cholesterol (CH), and low-density lipoprotein (LDL) levels, thereby causing atherosclerosis and cardiovascular disease [28, 31, 32]. Zinc is an important component of the antioxidant enzyme superoxide dismutase (Cu-ZnSOD) [32, 33]. The zinc ion concentration in the human body is an important parameter that helps regulate the antioxidant defense system of the body [34–36]. In the human body, the antioxidant activity of vitamin A depends on sufficient zinc ion concentration [37–39]. Vitamin E, another effective antioxidant, has many functions that overlap with zinc ions, including the maintenance of cell membrane stability, antioxidant function, and regulation of prostaglandins [38, 40]. Studies have shown that malabsorption of vitamin E is accompanied by a deficiency of zinc ions, thus indicating some interaction between the two nutrients [38, 41]. One possible explanation for the relationship between zinc ions, vitamins, and oxidation is that the body lacks zinc ions, which in turn leads to a decrease in the supply or utilization of vitamins A and E and ultimately leads to an increase in oxidation. This can create an imbalance in the ratio of oxidants to antioxidants (oxidative stress) in the body [41].

In addition, this reduction in antioxidant capacity indicates that LDL is more likely to be oxidized. The results of this study are consistent with those obtained by other researchers who observed low antioxidant levels in smokers; LDL in smokers is more likely to be oxidized. Early studies have shown the uptake of oxidized LDL cholesterol by monocytes and macrophages, forming foam cells, ultimately leading to atherosclerosis [42–44]. Therefore, the risk of atherosclerosis in diabetic patients is higher, not because of increased serum LDL levels but because of a higher likelihood of oxidized serum LDL. As the antioxidant concentration is reduced, oxidized LDL cholesterol is more likely to cause atherosclerosis [45, 46].

The serum zinc ion concentration that this study focuses on is a more accurate indicator for CHD detection and has a greater clinical application value than other indicators. In summary, this meta-analysis emphasized that low zinc ion concentration is related to the occurrence of CHD. For monitoring CHD, it is necessary to detect serum zinc ion concentration.

## 5. Conclusion

The results of this meta-analysis reveal that a decrease in serum zinc ion concentration is related to the occurrence of coronary heart disease. Clinically, monitoring the serum zinc ion levels is proven to be of great significance for patients with coronary heart disease.

## Figures and Tables

**Table 1:** Baseline information. The gold standard for diagnosing CHD in the experimental group is coronary angiography.

Id	Author	Country	Years	Is the method of diagnosing disease the gold standard	Types of test specimens for zinc ions	The mean value of zinc ion concentration in the control group	The standard deviation of zinc ion concentration in the control group	Number of the control group	Mean value of zinc ion concentration in the experimental group	The standard deviation of zinc ion concentration in the experimental group	Number of the experimental group
No. 1	Meng et al. [16]	China	2021	Yes	Serum	14.77 *μ*mol/L	2.86	1103	14.18 *μ*mol/L	2.87	1103
No. 2	Soinio et al. [21]	Finland	2007	Yes	Serum	15.8 *μ*mol/L	2.5	796	15.2 *μ*mol/L	2.5	254
No. 3	Basnet et al. [22]	Nepal	2020	Yes	Serum	13.7 mg	3.3	233	13.5 mg	2.6	233
No. 4	Cebi et al. [23]	Turkey	2011	Yes	Serum	0.91 *μ*g/dI	0.18	20	0.85 *μ*g/dI	0.55	30
No. 5	Hasanato [24]	Saudi Arabia	2020	Yes	Serum	15 *μ*mol/L	3.2	50	13.5 *μ*mol/L	1.9	50
No. 6	Islamoglu et al. [25]	Turkey	2011	Yes	Serum	0.67 ng/L	0.14	34	0.61 ng/L	0.16	33
No. 7	Li et al. [26]	China	1990	Partly	Serum	20.17 *μ*mol/L	3.97	31	14.39 *μ*mol/L	4.44	31

**Table 2:** Quality assessment form.

	No. 1	No. 2	No. 3	No. 4	No. 5	No. 6	No. 7	No. 8	No. 9	No. 10	No. 11	No. 12	Total score
Li et al. [26]	1	1	2	2	0	0	0	1	2	2	2	2	15
Rana-2020	2	1	2	1	0	0	0	2	1	2	2	2	15
Islamoglu et al. [25]	2	2	2	2	0	0	0	2	2	2	2	2	18
Soinio et al. [21]	2	2	2	2	0	1	0	2	2	2	2	2	19
Meng et al. [16]	2	2	2	2	0	0	0	2	2	2	2	2	18
Cebi et al. [23]	2	1	2	2	0	0	0	2	2	2	2	2	17
Basnet et al. [22]	2	2	2	2	1	1	1	2	2	2	2	2	21

## Data Availability

The literature data supporting this meta-analysis are from previously reported studies and datasets, which have been cited. The processed data are available from the corresponding author upon request.
